# Harmful Effect of *Rheinheimera* sp. EpRS3 (*Gammaproteobacteria*) Against the Protist *Euplotes aediculatus* (Ciliophora, Spirotrichea): Insights Into the Ecological Role of Antimicrobial Compounds From Environmental Bacterial Strains

**DOI:** 10.3389/fmicb.2019.00510

**Published:** 2019-03-22

**Authors:** Carolina Chiellini, Chiara Pasqualetti, Olivia Lanzoni, Camilla Fagorzi, Chiara Bazzocchi, Renato Fani, Giulio Petroni, Letizia Modeo

**Affiliations:** ^1^Department of Biology, University of Florence, Florence, Italy; ^2^Department of Biology, University of Pisa, Pisa, Italy; ^3^Department of Veterinary Medicine, University of Milan, Milan, Italy

**Keywords:** *Rheinheimera* sp. EpRS3, ciliate, TEM, FISH, culture experiment, endosymbiont, antimicrobial

## Abstract

*Rheinheimera* sp. strain EpRS3, isolated from the rhizosphere of *Echinacea purpurea*, is already known for its ability to produce antibacterial compounds. By use of culture experiments, we verified and demonstrated its harmful effect against the ciliated protist *Euplotes aediculatus* (strain EASCc1), which by FISH experiments resulted to harbor in its cytoplasm the obligate bacterial endosymbiont *Polynucleobacter necessarius* (*Betaproteobacteria*) and the secondary endosymbiont “*Candidatus* Nebulobacter yamunensis” (*Gammaproteobacteria*). In culture experiments, the number of ciliates treated both with liquid broth bacteria-free (Supernatant treatment) and bacteria plus medium (Tq treatment), decreases with respect to control cells, with complete disappearance of ciliates within 6 h after Tq treatment. Results suggest that *Rheinheimera* sp. EpRS3 produces and releases in liquid culture one or more bioactive molecules affecting *E. aediculatus* survival. TEM analysis of control (not treated) ciliates allowed to morphologically characterize both kind of *E. aediculatus* endosymbionts. In treated ciliates, collected soon after the arising of cell suffering leading to death, TEM observations revealed some ultrastructural damages, indicating that *P. necessarius* endosymbionts went into degradation and vacuolization after both Supernatant and Tq treatments. Additionally, TEM investigation showed that when the ciliate culture was inoculated with Tq treatment, both a notable decrease of *P. necessarius* number and an increase of damaged and degraded mitochondria occur. FISH experiments performed on treated ciliates confirmed TEM results and, by means of the specific probe herein designed, disclosed the presence of *Rheinheimera* sp. EpRS3 both inside phagosomes and free in cytoplasm in ciliates after Tq treatment. This finding suggests a putative ability of *Rheinheimera* sp. EpRS3 to reintroduce itself in the environment avoiding ciliate digestion.

## Introduction

*Rheinheimera* sp. strain EpRS3 is a *Gammaproteobacterium* belonging to the family *Chromatiaceae*. The strain was isolated from the rhizospheric soil of *Echinacea purpurea* (Chiellini et al., 2014), a medicinal plant previously described for its antimicrobial activity (Hudson, 2012). *Rheinheimera* EpRS3 shows resistance to several antibiotic compounds (Mengoni et al., 2014) and can inhibit the growth of various bacteria isolated from both *E. purpurea* rhizospheric soil and plant tissues (Maida et al., 2016). Additionally, EpRS3 can inhibit the growth of opportunistic human pathogenic bacteria belonging to the *Burkholderia cepacia* complex (Bcc) (Chiellini et al., 2017), as well as the growth of different bacterial pathogens (e.g., *Acinetobacter baumanni*, *Klebsiella pneumoniae*) the majority of which exhibit a multi-drug-resistance phenotype (Presta et al., 2017). It has been hypothesized that the production of antimicrobial compounds, and the subsequent antagonistic activity of some bacterial strains belonging to the same or different taxa, might play a (key) role in driving the structuring of microbial communities interacting with eukaryotic macro-organisms (Maida et al., 2016). The toxicity of bacteria belonging to *Rheinheimera* genus against other organisms has been also described in other strains such as GR5, showing antimicrobial activity against Gram-positive and Gram-negative bacteria, yeast, and algae (Chlorophyceae, Trebouxiophyceae) (Chen et al., 2010; Romanenko et al., 2015). Interestingly, *Rheinheimera* sp. strain GR5 can synthesize an l-lysine oxidase with antimicrobial activity associated with generation of hydrogen peroxide (Chen et al., 2010). Recently, the antibacterial toxicity of the marine bacterium *Rheinheimera japonica KMM 9513* has been described as well. This strain is able to exert the antimicrobial activities against *Bacillus subtilis* and/or *Enterococcus faecium* and *Staphylococcus aureus* by synthesizing phthalates and diketopiperazines (Kalinovskaya et al., 2017). Moreover, the anti-quorum sensing activity of the diketopiperazine factor cyclo (Trp-Ser) produced by *Rheinheimera aquimaris* QSI02 against *Chromobacterium violaceum* CV026 and *Pseudomonas aeruginosa* PA01 has been evidenced, focusing the attention on the potentiality of this bacterial genus to interact with other bacterial species in environmental microbial communities (Sun et al., 2016).

Under an ecological point of view, the production of bactericidal compounds from environmental bacterial strains can be related to different factors such as the competition for nutrients and the occupation of a specific niche by eliminating prior residents (Hibbing et al., 2010). On the other side, bacteria in the environment can produce molecules affecting the survival of eukaryotic organisms such as fungi. This is the case for example of some rhizospheric bacteria producing molecules with antifungal activity to protect the plant against pathogens (e.g., Pandey et al., 1997; Ligon et al., 2000; Cho et al., 2007). Interestingly, few studies focused the attention on bacterial antimicrobial compounds affecting the growth and survival of protists. An example is that of the environmental pathogenic *P. aeruginosa*, whose type three secretion system (TTSS) effector proteins allow the bacterium inducing necrosis and apoptosis, and killing of free-living *Acanthamoeba* species (Abd et al., 2008). Lovejoy et al. (1998) documented how *Pseudoalteromonas* sp. bacteria affect dinoflagellates survival, causing rapid cell lysis, and death of gymnodinoids (*Gymnodinium catenatum*) and raphidophytes (*Chattonella marina* and *Heterosigma akashiwo*). A deleterious effect of freshwater bacteria *Janthinobacterium lividum* and *C. violaceum* against flagellated protists *Ochromonas* sp. and *Spumella* sp. (Chrysophyceae) has also been described, by means of violacein production from bacterial strains (Matz et al., 2004b). In this scenario, a possible biocidal effect of *Rheinheimera* sp. EpRS3 against eukaryotic cells has become an intriguing topic. Could EpRS3 interact with eukaryotic unicellular microorganisms in complex microbial communities (e.g., biofilm)? Could this possible interaction determine the structuring of such communities in natural environments? To address these interesting questions, we performed experiments involving the ciliated protozoan *Euplotes aediculatus* (Ciliophora, Spirotrichea) monoclonal strain EASCc1, as a model organism to be tested in culture experiments with *Rheinheimera* sp. EpRS3.

The choice of *E. aediculatus* as model organism is based on different reasons such as the numerous previously published studies, providing a wide knowledge on the biological characteristics of this relatively easily cultivable ciliate (e.g., Sogin et al., 1986; Heckmann and Schmidt, 1987; Boscaro et al., 2012, 2013c; Vannini et al., 2017). Additionally, as symbioses involving ciliate hosts are widespread (e.g., see Vannini et al., 2010; Modeo et al., 2013b; Schrallhammer et al., 2013; Fokin et al., 2014, 2019; Castelli et al., 2018a; Lanzoni et al., 2019), fresh- and brackish-water strains of *E. aediculatus* are known to host in the cytoplasm the obligate bacterial endosymbiont *Polynucleobacter necessarius* (Heckmann and Schmidt, 1987; Vannini et al., 2005, 2007) and, possibly, also secondary symbionts (Boscaro et al., 2012), and the strain EASCc1 used for the present study made no exception. Indeed, from a preliminary fluorescence *in situ* hybridization (FISH) screening, *E. aediculatus* strain EASCc1 was known to host in its cytoplasm two bacterial symbionts: *P. necessarius* (*Betaproteobacteria*) as primary endosymbiont, and an unidentified *Gammaproteobacterium* as secondary endosymbiont.

The symbiotic relationship between the endosymbiont *P*. *necessarius* and its host *E. aediculatus* falls within obligate mutualism: the two involved species live in close proximity and interdependently with one another so that one cannot survive without the other. The symbionts had assumed an obligate role: after their removal by ciliate ampicillin treatment, ciliates cannot properly divide anymore and after one week they eventually die (Heckmann and Schmidt, 1987). Thus, the present study was conceived to verify by means of culture experiments, and FISH and TEM analyses:

(1) The possible deleterious effect of *Rheinheimera* sp. EpRS3 on this ciliate, taken as a model system among freshwater ubiquitous grazing protozoans, in case the deleterious effect occurred in few hours [i.e., *Rheinheimera* sp. EpRS3 bioactive molecule(s) also affect eukaryotic cells]; this effect has been herein defined as “direct” effect.
(2) The possible deleterious effect of *Rheinheimera* sp. EpRS3 on this ciliate, in case the deleterious effect occurred after about 1 day as a consequence of the depletion of the indispensable endosymbiont *P. necessarius* [i.e., *Rheinheimera* sp. EpRS3 bioactive molecule(s) only affect bacterial cells]; this effect has been herein defined as “not direct” effect.
(3) The possible resistance of *P. necessarius* to the presence of *Rheinheimera* sp. EpRS3 in case *E. aediculatus* was not affected at all (a kind of host protection effect like the one documented for *Acanthamoeba and Legionella:* Linder, 1999) [i.e., *Rheinheimera* sp. EpRS3 bioactive molecule(s) are shadowed by eukaryotic cells and do not affect host bacterial endosymbionts].

Both FISH and TEM investigation supported a direct deleterious effect of *Rheinheimera* sp. EpRS3 on *Euplotes* cells, with mitochondria being a probable target of the bioactive molecule(s). Interestingly, TEM analysis on affected *E. aediculatus* cells showed the presence of *Rheinheimera* sp. EpRS3 in *Euplotes* cytoplasm both inside and outside digestive vacuoles. A deleterious effect was also observed on the endosymbiont *P. necessarius*; the secondary endosymbiont, herein morphologically and molecularly characterized, appeared not affected.

## Materials and Methods

### *Euplotes aediculatus* Strain EASCc1 Isolation and Laboratory Maintenance

*Euplotes aediculatus* specimens were collected from the anaerobic side tank (0 ppt salinity) of the San Colombano activated sludge plant placed in Lastra a Signa (Florence, Italy), in November 2015. A single cell was isolated from the original population and was washed several times in sterile San Benedetto water (San Benedetto S. p. A. Italy) in order to minimize the presence of contaminating microorganisms. The cell was then accommodated in San Benedetto water and daily fed for 5 days with a drop of cerophyll medium (CM) inoculated with *Raoultella planticola* DSM3069 (*Gammaproteobacteria*, *Enterobacteriaceae*) to induce its exponential growth. CM is a commercially available mixture of pulverized wheat grasses. One liter of CM is composed by Cerophyll buffer (79,75 g/l Na2HPO4 and 27,1 g/l NaH2PO4), balanced salt solution (BSS = 20.8 g/l NaCl, 8 g/l MgSO4 × 7 H2O, 17 g/l MgCl2 × 6 H2O, 2,7 g/l CaCl2 × 2 H2O, and 4,6 g/l KCl), and stigmasterol (5.0 mg/ml in 99.8% ethanol). Food bacteria were stored at -20°C in 1 ml aliquots with 50% glycerin (v/v). Each aliquot was prepared in order to be sufficient to guarantee a good density of bacteria in 1 L of CM after 24 h of incubation at a temperature of 37°C. For the inoculation of CM, *R. planticola* aliquots were thawed and centrifuged for 5 min at 5,500 ×*g*. After discharging the supernatant, the pellet was washed and re-suspended with 1 ml of CM. The bacteria were centrifuged again for 3 min at 5,500 ×*g* to form a pellet. Lastly, they were resuspended in 1 ml of CM and then inoculated in 1 L of CM. Once *E. aediculatus* monoclonal strain EASCc1 was established, the culture was maintained in incubator at a temperature of 19 ± 1°C, with a 12:12 h irradiance of 200 μmol photons m^-2^s^-1^ (see Boscaro et al., 2013b and Castelli et al., 2018b for details). The obtained monoclonal culture was fed once a week with 1/4 bacterized CM of the total culture volume. In this culture conditions, ciliate doubling time was about 36 h. However, all ciliates used in the experiments were kept in starving conditions for 4 days before the experiment to ensure food digestion. The culture was constantly checked to ensure cell healthy conditions during the whole time of the experiments. *E. aediculatus* cell density was calculated under a stereo-microscope (Leica Wild M3C) using three technical replicates of 100 μl each; the cell number was estimated by mean, and calculated for 1 ml of culture (±150 cells/ml).

### Live Cell Imaging and Ciliate Identification

Living cells were immobilized on a slide for observation with the help of a coverslip without deforming them (Skovorodkin, 1990). Living cells were examined under differential interference contrast (DIC) using an Orthoplan Leitz microscope (Leitz, Germany) at ×300–1.250 magnifications, and photographed with a digital camera Canon S45 and True Chrome HDII. Ciliate identification was based on observations of morphological key-characters (i.e., cell shape and sizes, number and positions of cirri, length of buccal aperture, number and positions of dorsal kineties, type of argyrome on the dorsal surface) on an appropriate number of living individuals, and on the comparison of collected data with previous literature (Curds, 1975).

### DNA Extraction, Molecular Characterization of Ciliate Bacterial Secondary Endosymbiont and Sequencing of Almost Complete 16S rRNA Gene of *Rheinheimera* sp. EpRS3

From a preliminary FISH screening, *E. aediculatus* strain EASCc1 was known to host in its cytoplasm two bacterial symbionts: *P. necessarius* (*Betaproteobacteria*), primary endosymbiont, and an unidentified *Gammaproteobacterium*, secondary endosymbiont, so for the molecular characterization of the latter, about 50 starved *E. aediculatus* strain EASCc1 cells were washed several times in sterile distilled water and fixed in 70% ethanol. Total genomic DNA extraction was performed using the NucleoSpin^TM^ Plant II DNA extraction kit (Macherey-Nagel, Germany), following the protocol for mycelium suggested by the company. The 16S rRNA gene of the bacterial symbiont was amplified in a C1000TM Thermal Cycler (BioRad, Hercules, CA, United States) with the TaKaRa ExTaq (TaKaRa Bio Inc., Otsu, Japan). The16S rRNA gene of the *Gammaproteobacteria* endosymbiont was amplified using the primers alfa F19a (5′-CCTGGCTCAGAACGAACG-3′ Vannini et al., 2004) and R1492 (5′-GGNWACCTTGTTACGACTT-3′, modified from Lane, 1991). Each amplification consisted of 35 cycles of: a preliminary denaturation step at 94°C for 3 min, denaturation at 94°C for 30 s, annealing at 50°C for 30 s and elongation at 72°C for 2 min, and a final elongation step at 72°C for 6 min. In each PCR experiment, a control without DNA was always included. After evaluation by electrophoresis on 1% agarose gel, PCR products were purified with EuroGold Cycle-Pure kit (EuroClone1, Milan, Italy) and directly sequenced using prokaryotic universal primers, 16S R515ND (5′-ACCGCGGCTGCTGGCAC-3′), 16S F785ND (5′-GGATTAGATACCCTGGTA-3′), and 16S F343 (5′-TACGGGAGGCAGCAG-3′) (Vannini et al., 2004).

Genomic DNA of *Rheinheimera* sp. strain EpRS3 was obtained through thermal lysis. Briefly, an isolated colony (about 10^8^ bacterial cells after 12 h growth, Lodish et al., 2000) was picked up from an overnight culture in solid medium (in this case, TSA) and dissolved in 50 μl of saline solution (0.9% NaCl); bacterial cells were then submitted to thermal lysis in a thermoblock at 95°C for 10 min, followed by cooling on ice for 5 min. The supernatant obtained after 3-min centrifugation at maximum speed was then placed in a sterile tube and used as template DNA for amplification. The amplification of the 16S rRNA gene was performed using the same amplification protocol described for the ciliate symbiont, with universal prokaryotic primers F7 (5′-AGRGTTYGATYMTGGCTCAG-3′Vannini et al., 2004) and R1492 (5′-GGNWACCTTGTTACGACTT-3′, modified from Lane, 1991). After evaluation by electrophoresis on 1% agarose gel, PCR products were purified with EuroGold Cycle-Pure kit (EuroClone, Milan, Italy) and directly sequenced using prokaryotic universal primers, 16S R515ND (5′-ACCGCGGCTGCTGGCAC-3′), 16S F785ND (5′-GGATTAGATACCCTGGTA-3′), and 16S F343 (5′-TACGGG AGGCAGCAG-3′) (Vannini et al., 2004). The full-length sequence of the 16S rRNA gene of *Rheinheimera* sp. strain EpRS3 was necessary to design the specific FISH probe as described below.

### Probe Design for Detection of *Rheinheimera* sp. EpRS3 and FISH Analysis

A specific probe was designed *in silico* to detect *Rheinheimera* sp. EpRS3 using the almost complete characterized 16S rRNA gene sequence (Accession Number MH540131, this work). The specificity of probe Rhein443 (5′-TACCAACCCTTCCTCCTC-3′, Tm = 56°C) was tested *in silico* both on Ribosomal Database Project (Cole et al., 2013) and TestProbe tool 3.0 (SILVA rRNA database project, Quast et al., 2013). The probe was synthesized and labeled with Cy3 by Eurofins GMBH (Ebersberg, Germany), and the probe sequence was deposited in ProbeBase (Greuter et al., 2015). FISH experiments were carried out according to the protocol by Manz et al. (1992) and different formamide concentrations (0, 10, 20, and 30% v/v) were tested to find the optimal stringency level for the newly designed probe.

### Experimental Set-Up: Treatments of the Cultures

**FIGURE 1:**
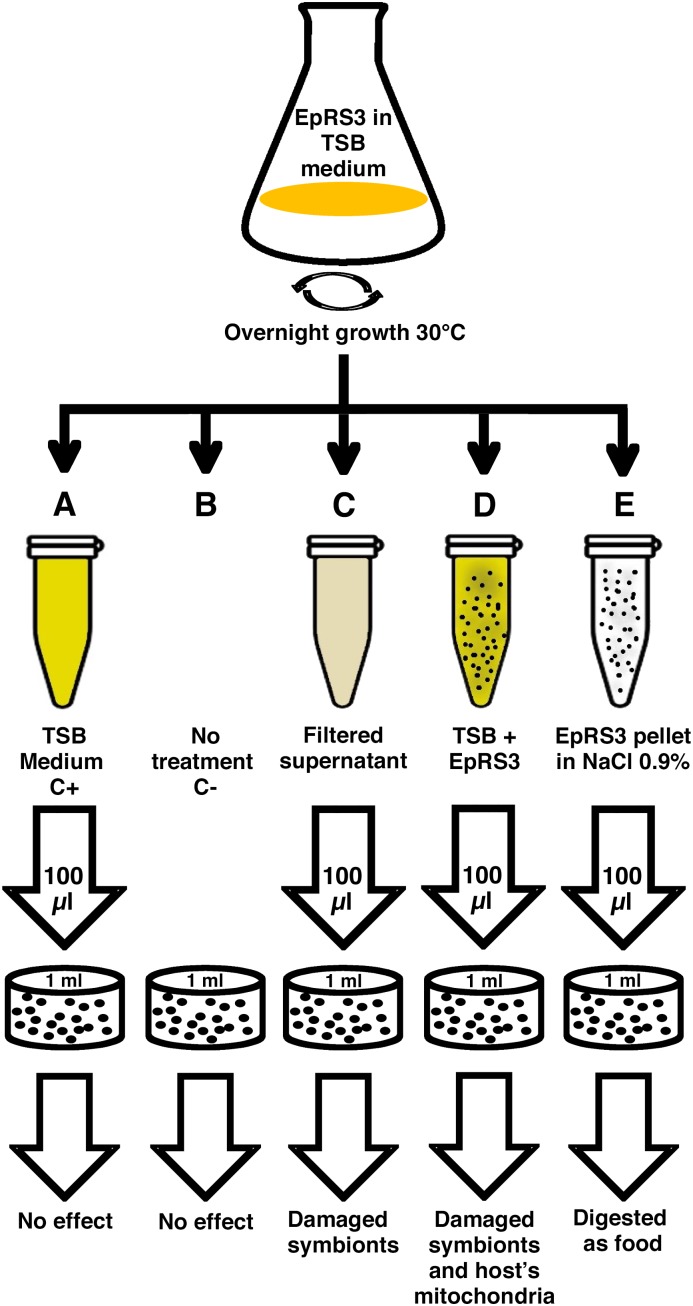
Schematic representation of the procedure followed for the experimental design.

The experimental procedure has been resumed in Figure 1. *Rheinheimera* sp. EpRS3 was grown overnight on tryptic soy broth medium (TSB) at 30°C in agitation. After growing, the liquid culture was immediately processed; five different treatments were prepared to treat five separate 1-ml ciliate sub-cultures of *E. aediculatus* strain EASCc1 consisting of 20–30 cells each (Supplementary Table S1). Previously, the ciliates were maintained in starving conditions for 4 days to let them complete digestion of their regular bacterial food (*Raoultella planticola*) and to minimize the presence of phagosomes with *Gammaproteobacteria* inside. All the sub-cultures received their specific treatment at the same time. These were the prepared treatments: (A) plain TSB medium (positive control); (B) absence of any treatment (negative control); (C) cell-free filtered supernatant from EpRS3 bacterial culture (“Supernatant” treatment); (D) liquid culture of EpRS3 bacterial cells with their growth TSB medium (“Tq” treatment); (E) EpRS3 cells without TSB growing medium, and resuspended in saline solution (0.9% NaCl in dH_2_O) (“Pellet” treatment).

Once each ciliate sub-culture was provided with its treatment, eukaryotic cells were counted at defined time points under the stereomicroscope (×10). The first count was performed immediately after the beginning of each treatment. Then cell counts were performed every 60 min, until 24 h. When *E. aediculatus* cells had showed evident signs of suffering, such as a clear swim deceleration, a prolonged absence of cell movement with ciliature still beating and/or cell number decrease, they were considered close to death and were collected and fixed for TEM and FISH analyses.

In order to study the possible cell damage that ciliates were facing, cell were fixed immediately before their death. A comparison with negative and positive control specimens was performed to highlight *Euplotes* cell structures affected by different EpRS3 treatments. Any potentially observed treatment effect on the ciliate obligate endosymbiont *P. necessarius* and on the secondary endosymbiont “*Ca.* Nebulobacter yamunensis” was recorded as well.

A total of ten replicate experiments were conducted in different days in order to confirm the harmful effect of *Rheinheimera* sp. EpRS3 vs. the ciliate *E. aediculatus* EASCc1. Once the effect was confirmed, an experiment was conducted with the aim to obtain fixed cells (treated and control ciliates) for FISH and TEM analyses: three different culture replicates, monitored up to 24 h, were performed to test the effect of the five different treatments reported in Figure 1 on the growth and survival of the *E. aediculatus* EASCc1. Variation in ciliate cell number was calculated in each case and expressed as % of T0 cell number (100%).

### Fluorescence *in situ* Hybridization (FISH) Analysis

Fluorescence *in situ* hybridization analysis was used both to assess the presence of the two different endosymbionts in *E. aediculatus* confirming preliminary observations using specific probes already available, and to verify the potential presence of *Rheineimera* sp. EpRS3 inside *E. aediculatus* EASCc1 cells which underwent culture experiments using the specific probe Rhein_443 (this work).

About 15 ciliate cells were randomly collected from both experimental and control cultures and were washed three times in sterile San Benedetto water and separately fixed on slides for FISH experiments as described by Szokoli et al. (2016).

Several different specific probes were used for FISH analysis (Table 1): Poly_862 (Vannini et al., 2005, labeled either with fluorescein or Cy3, green signal and red signal, respectively) to detect *P. necessarius*, NebProbe_203 (Boscaro et al., 2012, labeled with Cy3, red signal) to observe “*Ca.* Nebulobacter yamunensis,” and the newly designed specific probe Rhein443 (labeled with Cy3, red signal) to screen for *Rheinheimera* sp. EpRS3 presence. Two universal probes were employed as control to exclude the presence of other intracellular bacteria, namely Gamma_42 (Manz et al., 1992, labeled with Cy3, red signal) to detect *Gammaproteobacteria*, and Eub_338 (labeled with fluorescein or Cy3, green signal and red signal, respectively) targeting almost total Eubacterial diversity (Amann et al., 1990; Table 1).The presence of DAPI in the SlowFade^®^ Gold Antifade Reagent (Invitrogen) was used as additional control.

**Table 1:** List of probes used for FISH experiments reporting probe sequence, their fluorochrome, specificity, formamide concentration, and application.

Name	Sequence 5′ to 3′	Fluorochrome	Specificity	% Formamide in FISH experiment	Reference	Treatment
Eub_338	5′-GCTGCCTCCCGTAGGAGT-3′	Fluorescein	Almost 90% of known Eubacteria	0/15	Amann et al., 1990	C+ C- Supernatant Tq Pellet
NebProbe_203	5′-ATAGCGACTGCCCTAAAG-3′	Cy3	*“Ca.* Nebulobacter yamunensis*”*	15	Boscaro et al., 2012	C- C+
Poly_862	5′-GGCTGACTTCACGCGTTA-3′	Fluorescein cy3	*Polynucleobacter* genus	0	Vannini et al., 2005	C+ C-
Rhein443	5′-TACCAACCCTTCCTCCTC-3′	Cy3	*Rheinheimera* genus	0	Present work	C- C+ Supernatant Tq Pellet
Gamma_42	5′-GCCTTCCCACATCGTTT-3′	Cy3	*Gamma-proteobacteria* subclass	0	Manz et al., 1992	C- C+


*C+, TSB medium; C-, no treatment; Supernatant, filtered supernatant of *Rheinheimera* sp. EpRS3; Tq, EpRS3 + TSB; Pellet, EpRS3 in NaCl 0.9%.*

The pellet of *Rheinheimera* sp. EpRS3 from 2 ml, overnight liquid TSB culture (about 10^10^ cells/ml) was fixed in duplicate for FISH experiments using 2% PFA. 50 μl of the fixed bacterial pellet were dropped on the slide and hybridized according to Szokoli et al. (2016). FISH was performed using probe Rhein443 (labeled with Cy3, red signal) and Eub_338 (labeled with fluorescein, green signal) (Table 1) in order to verify the efficiency of the newly designed probe Rhein443 on *Rheinheimera* sp. EpRS3.

Images were obtained with a Leica DMR microscope, equipped with an HBO 50W/AC-L2 fluorescent lamp, a Leica DFC490 video camera, and a dedicated Software Leica IM1000 (v.1.0).

### Transmission Electron Microscopy (TEM)

Transmission electron microscopy analysis was used both to assess the morphology of the two different endosymbionts in *E. aediculatus*, and to verify the potential presence of *Rheineimera* sp. EpRS3 inside *E. aediculatus* EASCc1 cells which underwent culture experiments. For TEM analysis, eukaryotic cells sampled from each of the protist sub-cultures of culture experiments were fixed in a 1:1 mixture of 2% OsO_4_ in sterile distilled water and 2.5% glutaraldehyde in 0.2 M cacodylate buffer (pH 7.4); then cells were ethanol-dehydrated, transferred to 100% acetone, and embedded in an Epon araldite mixture (Modeo et al., 2013a).

After overnight growth in liquid TSB medium the pellet of *Rheinheimera* sp. EpRS3 (about 10^10^ bacterial cells/ml) was fixed for TEM analysis according to Nitla et al. (2018). Prokaryotic cells were fixed in 1.5-ml Eppendorf tubes and all the solutions (fixatives, ethanol, acetone, and Epon araldite embedding mixture) were directly added. At each step, the Eppendorf tube with prokaryotic cells was vortexed and centrifuged for 5 min at 5,500 *× g*; then, after discharging the supernatant, the next solution was added, and the pellet was re-suspended.

Ultrathin sections of both eukaryotic and prokaryotic cell TEM preparations were placed on copper grids and stained with uranyl acetate and lead citrate prior to observation with a JEOL 100S TEM.

### Hydrogen Peroxide Production by *Rheinheimera* sp. EpRS3

The production of hydrogen peroxide by *Rheinheimera* sp. EpRS3 was assessed with a colorimetric assay on agar medium based on the Prussian blue-forming reaction (Saito et al., 2007), that was used as described in Chen et al. (2010). The agar medium allows the identification of hydrogen peroxide-producing bacterial strains by the appearance of dark blue halos around the colonies, due to the formation of a precipitate. The Prussian blue agar is made as follows (1 L): 1 g of FeCl_3_⋅6H_2_O dissolved in 50 ml of distilled water (solution A); 1 g of potassium hexacyanoferrate(III) K_3_[Fe^III^(CN)_6_] dissolved in 50 ml of distilled water (solution B); preparation of 900 ml TSA medium; mixing solutions A and B, and pouring the mixture into TSA medium; the obtained medium, must be sterilized by autoclaving at 121°C for 20 min. Bacterial sample were streaked on Prussian blue TSA (PB-TSA), and incubated for 5 days at 30°C, until satisfactory growth; in parallel, different amounts of hydrogen peroxide were dropped on the same medium, and put under the same temperature conditions, in order to observe the blue precipitate formation.

## Results

### Ciliate Identification (Supplementary Figure S1)

Ciliate strain EASCc1 was confirmed in morphological inspections as *Euplotes aediculatus* as perfectly matching the description of the species (Curds, 1975); additionally, molecular analysis based on 18S rRNA gene sequencing confirmed the species assignation by morphological identification (C. Sigona, *pers. commun*.).

### Bacterial Molecular Characterization

The almost complete 16S rRNA gene sequence of the *Gammaproteobacteria* endosymbiont was achieved by direct sequencing after PCR on *E. aediculatus* cells. The sequence obtained was 1409 bp long (Acc. Number MH608339, this work) and was 100% identical with “*Ca*. Nebulobacter yamunensis” (GenBank HE794998) already present in NCBI database.

The *Rheinheimera* sp. EpRS3 strain 16S rRNA gene sequence was obtained by direct sequencing from the bacterial strain DNA. The sequence was 1455 bp long (Acc. Number MH540131, this work) and 98.27% identical with *Rheinheimera pacifica* (GenBank NR114230).

### Culture Experiments: Cell Counts (Figure 1, 2, and Supplementary Figure S2)

**FIGURE 2:**
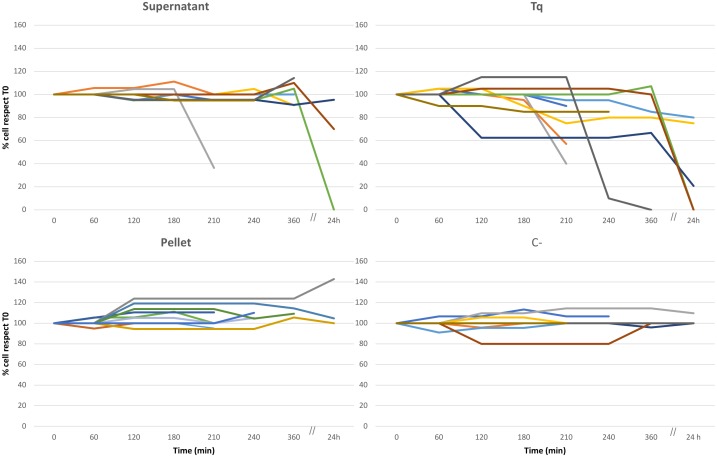
Culture experiment. Ten different replicates (indicated by ten different colors), three of them monitored over 24 h, performed to test the effect of *Rheinheimera* sp. EpRS3 on the growth, and survival of *E. aediculatus* EASCc1. Supernatant, Tq, Pellet, and C– treatments according to text. Variation in cell number is reported as % with respect to T0 cell number (100%).

The experimental procedure has been resumed in Figure 1. In all the replicate experiments, C+ and C- treatments (i.e., the treatment with plain bacteria TSB medium and the absence of any treatment, respectively) did not affect the overall health status of ciliates (Figure 2 and Supplementary Figure S2). However, several of the other treatments did affect ciliate survival (Figure 2). Supernatant treatment showed conflicting results depending on the different replicate: in two out of ten replicates, cell number dramatically decreased between 6 and 24 h from T0 (beginning of the treatment); moreover, cells stopped moving 2–4 h from T0. This behavior, with the same timing, was also recorded in Tq-treated ciliates. In a third replicate cell number remained constant over time, suggesting that there was not any effect on cell survival. Tq treatment impacted ciliates in all the ten replicates. After 2–4 h from T0, all ciliates stopped movement and showed cell suffering; in a single replicate ciliate number started a dramatic decreasing already after 1 h from T0. In all the replicates, the almost complete disappearance of treated ciliate sub-culture was recorded between 6 and 24 h.

Some Tq-treated cells were isolated soon after stopping movement and transferred to pure freshwater in order to roughly test whether the treatment effect was immediate and/or permanent. They apparently recovered well after some minutes, but it is not possible to assert if they were able to survive for a longer time and also to divide in the light of the TEM observation which reported on heavy *Euplotes*/endosymbiont cell damages (see later).

Pellet treatment did not affect protozoan survival in any of the ten replicate experiments, and the ciliate sub-culture survived over time: cells showed a healthy aspect, and, in some cases, they duplicated with an increment of number.

### Rhein_443 Probe Design

In the present study the probe Rhein_443 (5′-TACCAACCCTTCCTCCTC-3′, Tm = 56°C) was tested for its optimum formamide concentration by applying formamide from 0 to 30%, and the highest signal intensity was recorded at 0%. Its specificity was verified *in silico* both on Ribosomal Database Project (Cole et al., 2013) and on TestProbe tool 3.0 (SILVA rRNA database project Quast et al., 2013). The newly designed probe recognized *in silico* four non-target sequences, but its specificity was always verified during FISH experiments using the universal probe Gamma_42 targeting *Gammaproteobacteria* (Table 1).

### FISH Experiments (Figure 3 and Supplementary Figures S1, S3, S4)

**FIGURE 3:**
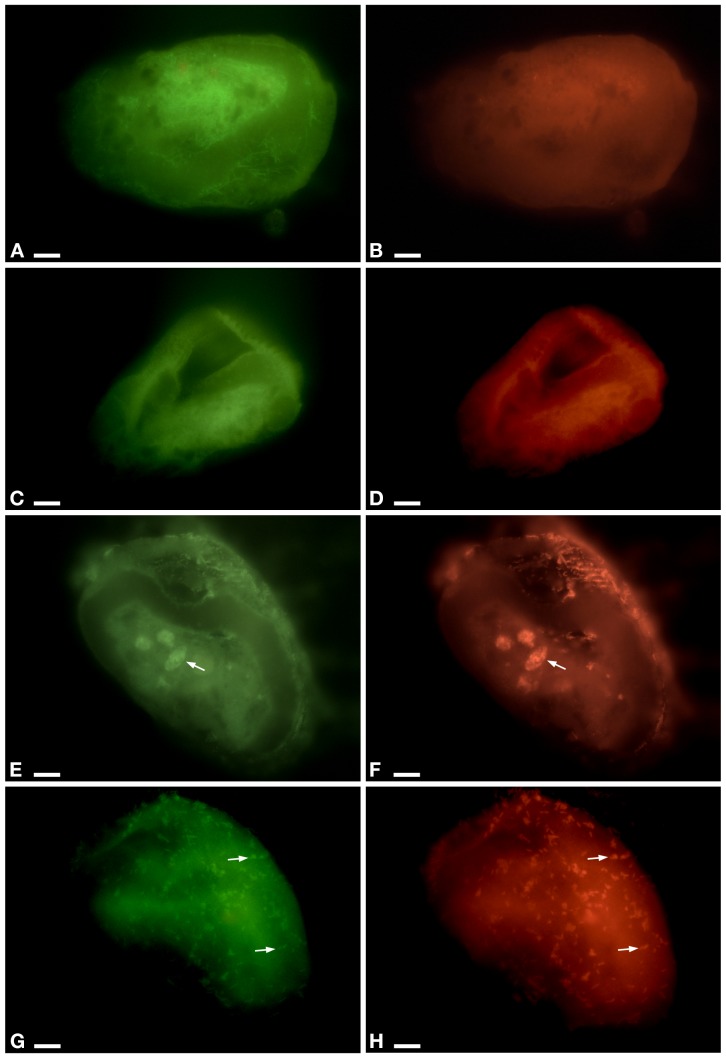
FISH experiment on *E. aediculatus* EASCc1 cells (0% formamide) using Eub_338 in green (detected almost 90% of known Eubacteria) and Rhein443 in red (detected *Rheinheimera* genus). **(A**,**B)** Negative control of culture experiment (i.e., not-treated ciliates). **(C**,**D)** Supernatant treatment. **(E**,**F)** Pellet treatment. Arrow, phagosome. **(G**,**H)** Tq treatment: signals of Rhein443_cy3 probe corresponding to *Rheinheimera* sp. EpRS3 cells (arrow). Scale bars: 10 μm.

In the cytoplasm of the ciliate, the endosymbiont *P. necessarius* was well visible in culture experiments under the light microscope due to its large size and high abundance (Supplementary Figures S1A,B). Preliminary FISH experiments on the ciliates with Poly_862, Gamma_42, and NebProb_203 probes highlighted the presence in the cytoplasm of two distinct endosymbiotic bacteria: the betaproteobacterium *P. necessarius* (Supplementary Figures S1C,D) and the gammaproteobacterium “*Ca.* Nebulobacter yamunensis” (Supplementary Figures S1E,F). FISH experiments on *Rheinheimera* sp. EpRS3 bacterial cells produced positive signals with both EUB_338_Fluo (Supplementary Figure S3A) and Rhein443_cy3 (Supplementary Figure S3B) probes. FISH experiments on C- and C+ ciliate subcultures hybridized with Poly_862, Gamma_42, and NebProb_203 probes, confirmed the presence of the two bacterial endosymbionts in ciliate cytoplasm. On the contrary, FISH experiments on the same two ciliate subcultures with Eub_338 and Rhein443 gave positive signals only with the first probe, revealing the absence of *Rheinheimera* sp. EpRS3 cells (C-, Figure 3A,B; C+, data not shown).

In Supernatant-treated sub-culture ciliates Rhein443 probe gave negative signal indicating the absence of *Rheinheimera* sp. EpRS3 (Figure 3C,D). In Pellet-treated ciliates, Rhein443 probe highlighted the presence of *Rheinheimera* sp. EpRS3 inside large food vacuoles (Figure 3E,F).

On ciliates of the Tq-treated subculture, Rhein443 probe revealed positive signals in the cytoplasm, indicating the presence of *Rheinheimera* sp. EpRS3 (Figure 3G,H). It is worth noting that these positive signals indicated the presence of *Rheinheimera* sp. EpRS3 both in small phagosomes and free in the cytoplasm, i.e., not included in phagosomes. Additionally, some *Rheinheimera* sp. EpRS3 bacteria were targeted outside the ciliate (Supplementary Figure S4), divergently from what observed in all other FISH experiments.

### TEM Observation (Figure 4–8)

**FIGURE 4:**
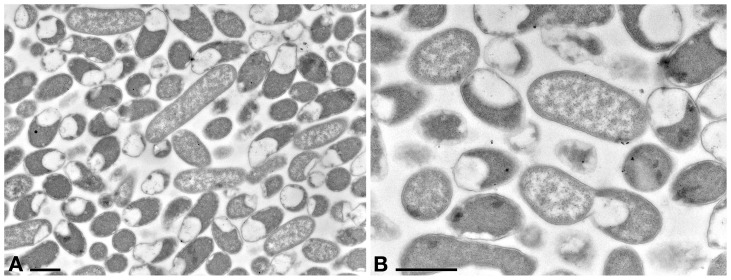
TEM observation on the pellet of *Rheinheimera* sp. EpRS3 processed after overnight liquid TSB culture (stationary growth phase). **(A)** Cells show different size and different aspect due to pellet sectioning. **(B)** Some cells are dividing. Scale bars: 1 μm.

A preliminary TEM experiment was carried out on *Rheinheimera* sp. EpRS3 in order to obtain the first morphological-ultrastructural data on these bacterial cells (Figure 4). Cells generally appeared rod-shaped and encircled by a double membrane. They showed a linear outline and a highly electron-dense peripheral region; cell electron-density decreased in the inner structure, leading to a clear central core. Cells were fixed during their stationary growth phase and did not show the presence of different bacterial morphologies, which might occur with diverse life cycle stage. No bacterial flagellum was observed; however, the lack of flagella could also be due to TEM procedure. Bacterial cell dimensions ranged from ∼ 1.5 μm × 0.8 μm up to ∼ 4 μm × 0.8 μm.

**FIGURE 5:**
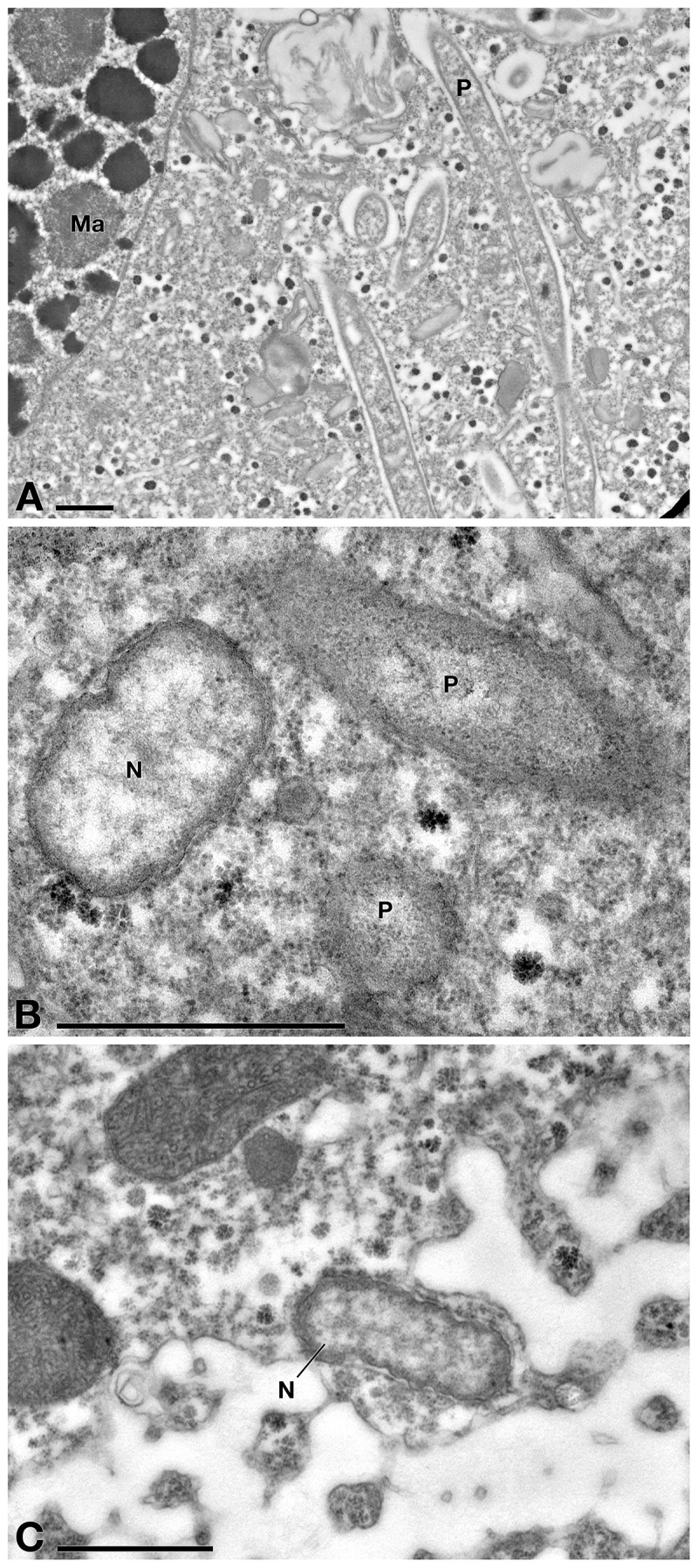
TEM observation on *E. aediculatus* EASCc1 cytoplasm without treatment (C– sub-culture specimens). **(A)** Some *Polynucleobacter necessarius* (P) endosymbionts adjacent to macronucleus (Ma). **(B)** “*Ca.* Nebulobacter yamunensis” (N) and *P. necessarius*. **(C)** Another specimen of “*Ca*. Nebulobacter yamunensis.” Scale bars: 1 μm.

TEM observation on *E. aediculatus* cells confirmed the results of FISH experiments. As expected, in C- cells (no treatment) the presence of two endosymbionts was observed, i.e., *P. necessarius* (maximum size: ∼ 6.5 μm × 0.4 μm; very abundant) (Figure 5A) and “*Ca.* Nebulobacter yamunensis” (size: ∼ 1.5 μm × 0.6 μm; rare) (Figure 5B,C). Both the endosymbionts showed a double membrane as typical of Gram-negative bacteria. *P. necessarius* cells were characterized by a slender aspect, an undulating external membrane, a non-homogeneous, generally electron dense cytoplasm, with some inner clearer regions where the presence of nucleoids sometimes is visible. “*Ca.* Nebulobacter yamunensis” usually appeared squatter and showed a homogeneous, clear inner cytoplasm, which becomes more electron dense in its periphery. A clear halo was sometimes visible around *P. necessarius* cells.

**FIGURE 6:**
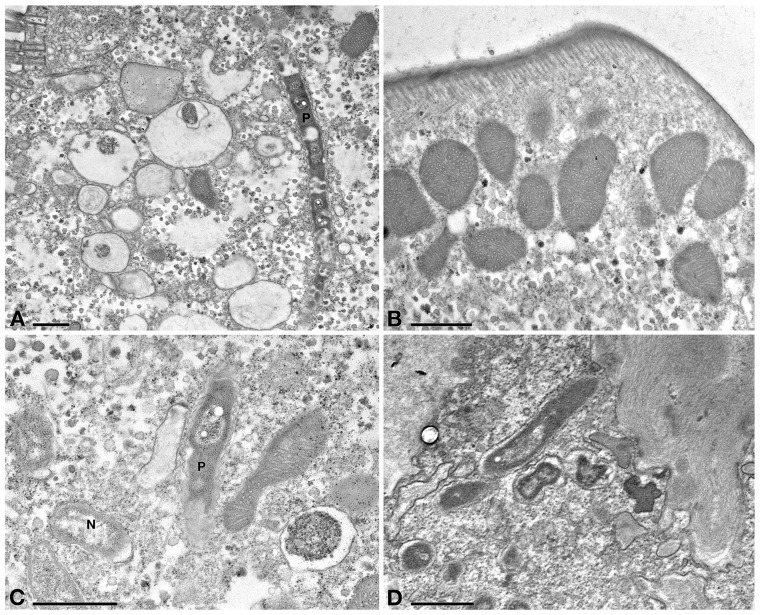
TEM observation on *E. aediculatus* EASCc1 treated with Supernatant treatment. (**A**) Ciliate cytoplasm vacuolized and a degraded *P. necessarius* (P). **(B)** Plasma membrane and mitochondria appear regular. **(C)** After Supernatant treatment “*Ca.* Nebulobacter yamunensis” (N) is not affected, while *P. necessarius* is affected (P). **(D)** Some *P. necessarius* endosymbionts at different stages of degradation. Scale bars: 1 μm.

Positive control cells (C+, treatment with sterile TSB medium) showed no ultrastructural differences with C- cells (data not shown), as bacteria growth medium itself did not affect ciliate cells in any possible way. By TEM observation, *E. aediculatus* cells treated with Supernatant treatment (*Rheinheimera* sp. EpRS3 cell-free supernatant), showed a slightly vacuolized cytoplasm (Figure 6A,B), while mitochondria and plasma membrane appeared not affected (Figure 6C). Ciliates still hosted in the cytoplasm many *P. necessarius* bacterial endosymbionts, but several of them showed clear suffering signs, such as high cytoplasm vacuolation and degradation, being altered at different grades (Figure 6A,C,D). Representatives of “*Ca.* Nebulobacter yamunensis” generally showed a rather regular low abundance and appearance (Figure 6B).

Several very electrodense bodies enclosed in vacuoles were observed in the cytoplasm of both Supernatant (Figure 6D) and Tq (see later) treated cells; these are obliquely- or cross-sectioned, highly degraded *P. necessarius* cells, as their abundance and general appearance fit with those of longitudinally sectioned, degraded *P. necessarius* endosymbionts observed in the same TEM sections.

**FIGURE 7:**
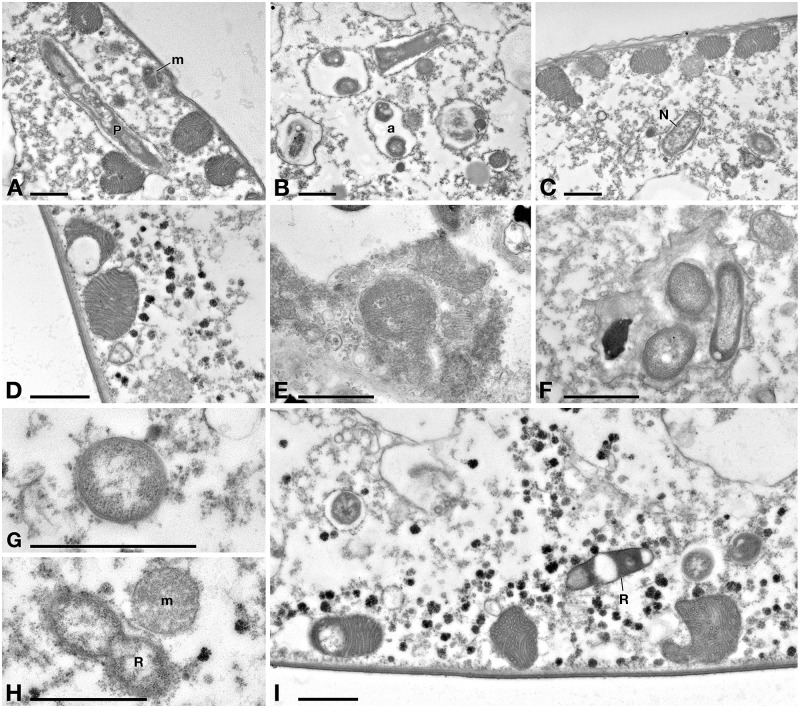
TEM investigation on *E. aediculatus* EASCc1 after Tq treatment. **(A)** Degraded mitochondria (m) and a vacuolized, highly degraded *P. necessarius* (P) in longitudinal section. **(B)** *P. necessarius* cells included in autophagosomes (a). **(C)** “*Ca.* Nebulobacter yamunensis” (N) and some damaged mitochondria. (**D**,**E)** Broken, highly degraded mitochondria. **(F)** *Rheinheimera* sp. EpRS3 cells (R) inside a phagosome. **(G)** *Rheinheimera* sp. strain EpRS3 free in ciliate cytoplasm. **(H)** A dividing specimen of *Rheinheimera* sp. (R) and a degraded mitochondrion (m). **(I)** *Rheinheimera* sp. free in cytoplasm in longitudinal section (R). Scale bars: 1 μm.

In Tq-treated ciliates, plasma membrane was still well conserved, while majority of *P. necessarius* endosymbionts were vacuolized and degraded as much as in Supernatant-treated cells. (Figure 7A). Autophagosomes were also present in the cytoplasm, in some cases including *P. necessarius* cells (Figure 7B). The endosymbiont “*Ca.* Nebulobacter yamunensis” was apparently not affected by the treatment (Figure 7C). Unlike what observed in Supernatant-treated cell, in Tq-treated ciliates mitochondria were very degraded as well; many of them were broken and, in some cases, they appeared to have undergone a kind of membrane disassembly (Figure 7A,B,D).

In Tq treated-cells, EpRS3 bacterial cells were observed both enclosed (Figure 7F) and not enclosed in phagosomes (i.e., free in the cytoplasm) (Figure 7G–I). They could be identified based on shape, dimensions, and appearance that distinguished them both from *P. necessarius* and “*Ca.* Nebulobacter yamunensis” endosymbionts. These results confirmed FISH experiment findings.

**FIGURE 8:**
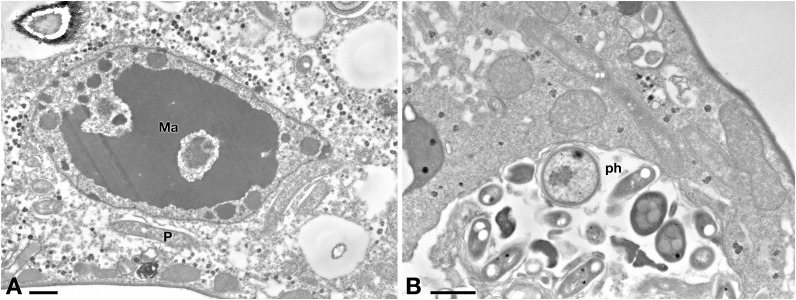
**(A)** TEM investigation on *E. aediculatus* EASCc1 after Pellet treatment. Cell aspect resembles that of C– cells. Ma, macronucleus; P, *P. necessarius* endosymbiont. **(B)** A large phagosome (ph) with bacteria undergoing digestion. Scale bars: 1 μm.

Ciliate cells treated with Pellet treatment showed cytoplasm and endosymbiont appearance similar to C- cells (Figure 8A,B). In Pellet-treated ciliates many *Rheinheimera* sp. EpRS3 cells (R) were observed inside phagosomes, in different digestion stages (Figure 8B).

### Hydrogen Peroxide Production by EpRS3 (Supplementary Figure S5)

Prussian blue agar medium assay revealed that *Rheinheimera* EpRS3 was able to grow on the modified PB-TSA medium. The growth after 5 days incubation did not reveal any hydrogen peroxide production by *Rheinheimera* EpRS3; this was evidenced by the absence of the blue halo around bacterial cells (Supplementary Figure S5, right side). This was also confirmed through comparison with the blue halos appeared around hydrogen peroxide dropped at different concentration on the same modified medium (Supplementary Figure S5, left side).

## Discussion

Antagonistic interactions in complex microbial communities have been largely investigated in the past years as means of competition for space and resources (e.g., Hibbing et al., 2010; Becker et al., 2012). Antagonistic interactions among bacterial species are often expressed by means of the release of toxic/antagonistic compounds, that are used to prevent the entrance of other bacterial microorganisms, by creating an inhospitable zone for competitors (Stubbendieck and Straight, 2016; Stubbendieck et al., 2016). Bacteria are largely studied for their ability to produce metabolites with a biocidal effect against other bacteria (Maida et al., 2016; Chiellini et al., 2017) and against fungi (Magnusson et al., 2003). Antagonistic interactions between bacteria and protists, are instead a still poorly investigated, but ecologically relevant field (Matz et al., 2004a). The ecological role of this ability might find its explanation as an attempt to avoid protist grazing, and help improving the survival of bacterial population in many environments (e.g., Jousset et al., 2006).The strategies to survive against protist predation include the production of bioactive metabolites (Matz et al., 2004a), bacterial motility characteristics (Matz and Jürgens, 2005), the biological properties of the cell external surface (Wildschutte et al., 2004), and the communication among cells (Matz et al., 2004a).

In the present research, the antagonistic interaction between the freshwater ciliated protozoan *E. aediculatus* strain EASCc1 and the antimicrobial producer endophytic *Rheinheimera* sp. EpRS3 has been documented in culture experiments, and confirmed for the first time by means of FISH and TEM experiments. Firstly, the lack of any variation in protists cell number observed in the culture experiments (C+ and C- treatments) allows to hypothesize that all the effects highlighted in ciliates treated with the other three treatments (Supernatant and Tq) were due to the presence of *Rheinheimera* sp. EpRS3 bacteria and to one or more putative bioactive molecule(s) they produce (Figure 2). Moreover, the positive signals of Gamma, Poly, and NebProb FISH probes in the cytoplasm of *E. aediculatus* cells provided to C+ and C- treatments (data not shown), also confirmed that the bacterial endosymbionts were not affected by either the TSB medium or the cell manipulation occurred during the experimental procedures.

In FISH experiments, the absence of signal by the probe Rhein443 in *E. aediculatus* cells of C- (Figure 3B), C+ (data not shown), and Supernatant (Figure 3D) treatments, and the positive signals observed after Pellet and Tq treatments (Figure 3F,H), indicate the presence of living *Rheinheimera* sp. EpRS3 cells only in ciliates treated with the latter two treatments. After Tq treatment, at difference with Pellet treatment where bacteria appear enclosed in phagosomes and in different digestion stages (Figure 3F), only a few bacterial cells are enclosed in small phagosomes, while most of them are free in the cytoplasm of treated ciliates (Figure 3H). These results have been confirmed by TEM observation (Figure 7G–I) and are in accordance with previous studies disclosing that some bacteria are capable of surviving to ciliate digestion in food vacuoles (King et al., 1988). In particular, *Gammaproteobacteria* and *Alphaproteobacteria* are reported as prevalent as digestion-resistant bacteria in ciliated protozoa (Gong et al., 2016). It could be hypothesized that *Rheinheimera* sp. EpRS3 cells can take advantage of suffering ciliates and easily escape phagosomes, thus entering the cytoplasm and starting to produce bioactive molecule(s) with a biocidal effect. Interestingly, also in Tq-treated *Euplotes* samples observed at TEM, dividing *Rheinheimera* sp. EpRS3sp. specimens have been observed free in ciliate cytoplasm, which indicates that these bacteria are healthy and might potentially colonize the host cytoplasm as an endosymbiont. However, ciliate mitochondrial degradation has been observed at TEM in Tq-treated *Euplotes*, which could be explained by the effect of *Rheinheimera*’s bioactive molecule(s). It can be speculated that these might be produced during the entrance of *Rheinheimera* cells in the ciliate cytoplasm, or before the entrance of the bacterium in the suffering ciliates. It cannot be ruled out the possibility that some autophagy phenomena concerning organelles such as mitochondria occurred in ciliate cytoplasm as well (Szokoli et al., 2016). Moreover, according to probe signals in FISH experiments, *Rheinheimera* sp. EpRS3 could possibly leave the ciliate when the latter is suffering, as suggested by the positive FISH signals of Rhein443_cy3 probe observed on ciliate surface (Supplementary Figure S4). This interesting result, if validated by further data, could support the hypothesis that healthy bacterial cells inside ciliates might perhaps reintroduce themselves into the environment, as it is known for other infectious ciliate endosymbionts (e.g., *Holospora*) (Potekhin et al., 2018).

Concerning the endosymbionts of *E. aediculatus*, after Supernatant and Tq treatments, *P. necessarius* (highly abundant endosymbiont) appeared very damaged (Figure 6A,C,D, 7A,B) while “*Ca.* Nebulobacter yamunensis” (less abundant endosymbiont) seemed not affected (Figure 6C, 7C) in TEM-processed ciliates. The symbiotic relationship between *Euplotes* and *Polynucleobacter* is well-known, as it is an obligate mutualistic endosymbiont (Heckmann, 1983; Heckmann and Schmidt, 1987; Görtz and Schmidt, 2004), while up to now nothing is known about the nature of the symbiotic association between *Euplotes* and “*Ca.* Nebulobacter yamunensis” as the latter has been only molecularly characterized (Boscaro et al., 2012). This is the first time the ultrastructure of this bacterium has been investigated and the present study gives hints on the not strict bound between the ciliate host and “*Ca.* Nebulobacter yamunensis,” as the endosymbiont is not affected by ciliate suffering.

In the light of the present findings, the following hypothesis can be formulated to explain *Rheinheimera* sp. EpRS3 behavior in the presence of the protist ciliate *E. aediculatus*. *Rheinheimera* EpRS3 sp. produces one or more different molecule(s) with an evident deleterious effect on *E. aediculatus*; these molecules are only present in the Tq treatment (bacteria plus culture medium). The Pellet treatment does not contain any trace of these bioactive molecules with deleterious potential; indeed, EpRS3 cells are ingested and digested by the ciliate together with food bacteria (*R. planticola*) without any apparent problem. In this case, *Rheinheimera* is treated as a food source by the protist, which can survive. On the other side, the filtered Supernatant, free of bacterial cells, is not sufficient to strongly affect the whole *E. aediculatus* population survival. This is demonstrated (i) by the cell counts on *E. aediculatus* repeated culture experiments, showing that only in a few cases the cell number decreases and (ii) by the TEM observation showing vacuolization and degradation of some of the *P. necessarius* endosymbionts, but no damage of fundamental ciliate organelles such as mitochondria. Finally, Tq treatment helped to highlight a possible explanation about the harmful effect exerted by EpRS3 on *E. aediculatus. Rheinheimera* sp. cells are initially ingested and stored in the phagosomes, treated as a food source by the protozoa. But the Tq treatment, might contain also all the bioactive molecules that are produced from the bacterium after overnight growth, which might damage host mitochondria leading *Euplotes* and its mutualistic symbiont to death. It is known that symbiotic *P. necessarius* has a very reduced genome and depends on its host for many metabolic pathways (Boscaro et al., 2013a). This could explain why *P. necessarius* suffers in the Tq treatment in presence of degraded mitochondria, while in the Supernatant treatment the bacterial endosymbionts decline probably due to the presence of bioactive molecules, not affecting host mitochondria.

The proposed scenario might be part of all those strategies used by bacteria in order to survive against protist predation, asserted through grazing pressure. Although further experiments should be performed to assert it with certainty, our data suggested that the toxicity of *Rheinheimera* EpRS3 might not be explained by means of production of hydrogen peroxides, contrary to what has already been reported in other *Rheinheimera* strains showing similar effects on bacteria and eukaryotes (Chen et al., 2010). The described hypothesis might be included among those strategies previously described as “post-ingestion adaptations” (Matz and Kjelleberg, 2005). In this case, the internalized bacterial cells are enclosed in digestive vacuoles, and face with an extremely acidophilic environment, as well as with the presence of degradative enzymes to survive. Sometimes, the resistance of bacteria in such hostile environment can be attributed to peculiar bacterial protective features such as in the case of the microbial S-layers of *Synechococcus* cells in the ciliate *Tetrahymena* (Koval, 1993). In this case, further investigation will be required to find out molecular mechanisms related to the resistance of the bacterium inside digestive vacuoles.

The present study is only preliminary, and the speculative hypotheses here proposed definitely need further investigation to be confirmed. We are planning to address the attention on the possible characterization of the bioactive molecule(s) produced by the bacterium by means of an integrated multidisciplinary study approach. On the light of the available literature, an interesting and still unexplored field is represented by the putative role of the *Rheinheimera* sp. EpRS3 TolC in the mechanisms leading to the interaction with the protozoan cell, as previously documented in *Legionella pneumophila* toward *Paramecium tetraurelia* (Nishida et al., 2018). Indeed, it has been previously demonstrated that EpRS3 genome harbors several genes encoding for components of efflux pump systems, like AcrB, belonging to the AcrAB/TolC system, or MdtB and MdtC, these last forming a heteromultimer complex, a subunit of MdtABC-TolC efflux pump (Presta et al., 2017). This information will help in elucidating the molecular mechanisms leading to the eukaryotic cell structures damage, to protist death, and to the possible re-entry of *Rheinheimera* sp. EpRS3 into the environment.

## Author Contributions

CC, CP, LM, GP, and RF designed the research. CC and CP performed the culture experiments. CP and LM performed the TEM experiments. CP and OL performed the FISH experiments. CC, CP, OL, and LM interpreted the results and wrote the manuscript. GP, RF, CB, and CF revised the manuscript. GP, CB, and RF financed the research. All authors critically read and approved the manuscript.

## Conflict of Interest Statement

The authors declare that the research was conducted in the absence of any commercial or financial relationships that could be construed as a potential conflict of interest.
