# Supplementary material for: Harmful Effect of Rheinheimera sp. EpRS3 (Gammaproteobacteria) Against the Protist Euplotes aediculatus (Ciliophora, Spirotrichea): Insights Into the Ecological Role of Antimicrobial Compounds From Environmental Bacterial Strains
Source: Front Microbiol. 2019 Mar 22;10:510. doi: 10.3389/fmicb.2019.00510 (PMC6457097; doi:10.3389/fmicb.2019.00510)

Supplementary Figure S1: Light microscopy (A, B) and FISH experiment (C - F) on *E. aediculatus* EASCc1 (C, D, E: 0% Formamide; F: 15% formamide). **A.** General view of a living cell. **B.** The cytostome with many *Polynucleobacter necessarius* (arrow) and few "*Ca.* Nebulobacter yamunensis" (arrowhead). **C.** Positive signals from numerous bacteria inside the host cytoplasm targeted by the probe EUB338_Fluo detecting almost 90% of known Eubacteria (arrow). **D.** Positive signals from numerous bacteria inside the host cytoplasm targeted by the probe Poly_862_cy3, specific for *Polynucleobacter* genus (arrow). **E.** Positive signals both in cytoplasm and inside phagosomes, highlighting the presence of *Gammaproteobacteria* targeted by the probe Gamma_42 (arrow). **F.** *Gammaproteobacteria* targeted by the probe NebProbe_203 specific for "*Ca.* Nebulobacter yamunensis" (arrow). Scale bars: 10 µm.

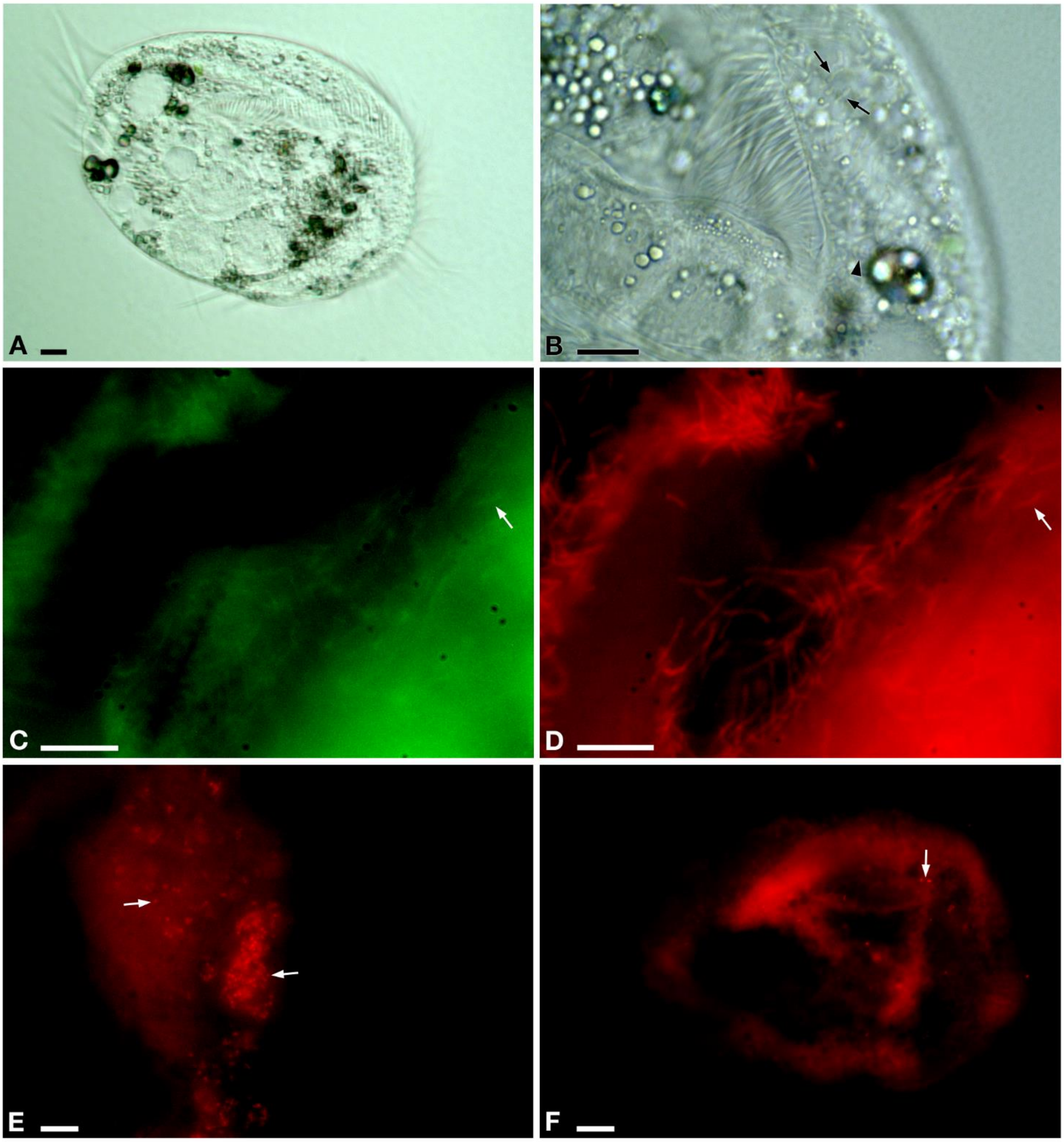

Supplementary Figure S2: Culture experiment. Five different replicates (indicated by five different colors), monitored over 210 minutes, performed to test the effect of TSB medium (C+) on the growth and survival of *E. aediculatus* EASCc1. Variation in cell number is reported as % with respect to T0 cell number (100%).

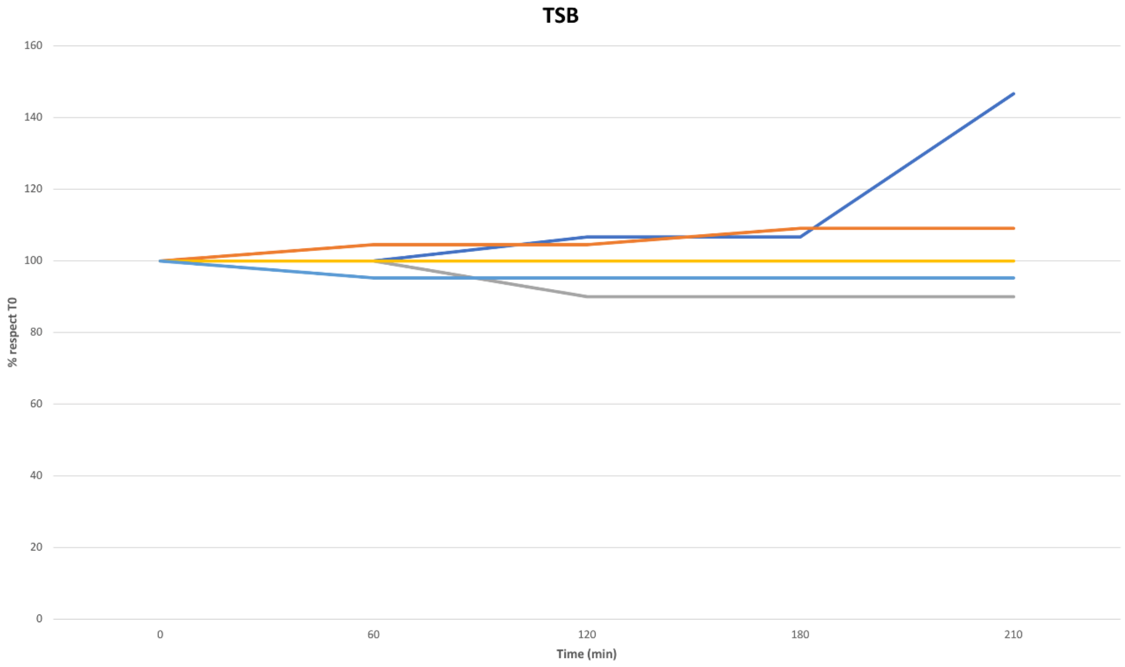

Supplementary Figure S3: FISH experiment on *Rheinheimera* sp. EpRS3 (0% formamide). **A.** Positive signals from numerous free-living bacteria targeted by the probe EUB_338_Fluo. **B.** Positive signals from bacteria targeted by the probe Rhein443 cy3 specific for *Rheinheimera* genus. Scale bars: 10 µm.

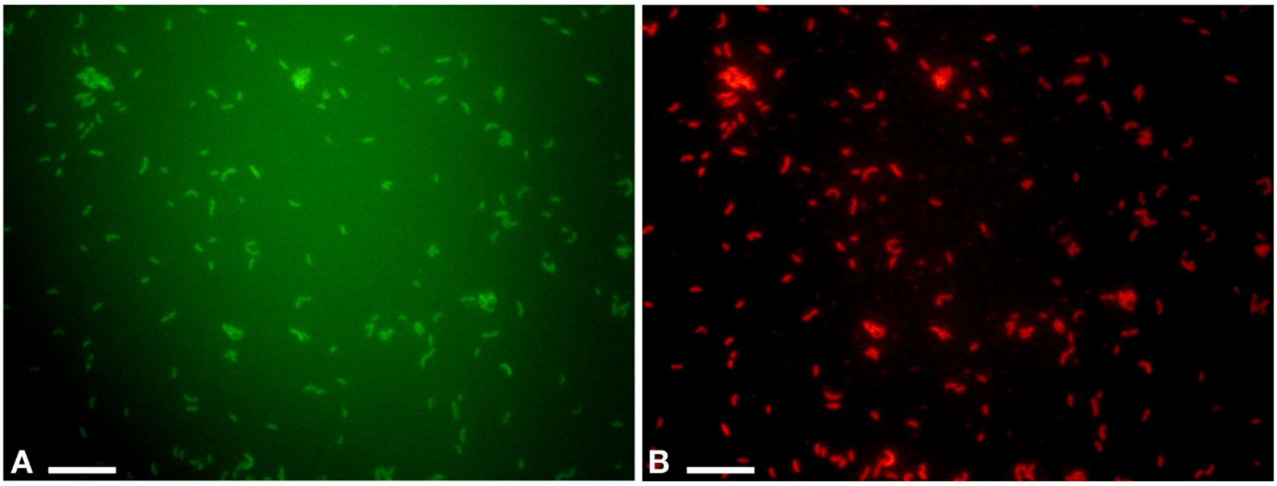

Supplementary Figure S4: FISH experiment on Tq-treated *E. aediculatus* EASCc1 cells (0% formamide) using probe Rhein443 in red (targeting *Rheinheimera* genus): signals of Rhein443_cy3 probe corresponding to *Rheinheimera* sp. EpRS3 cells on ciliate surface. Scale bar: 10 µm.

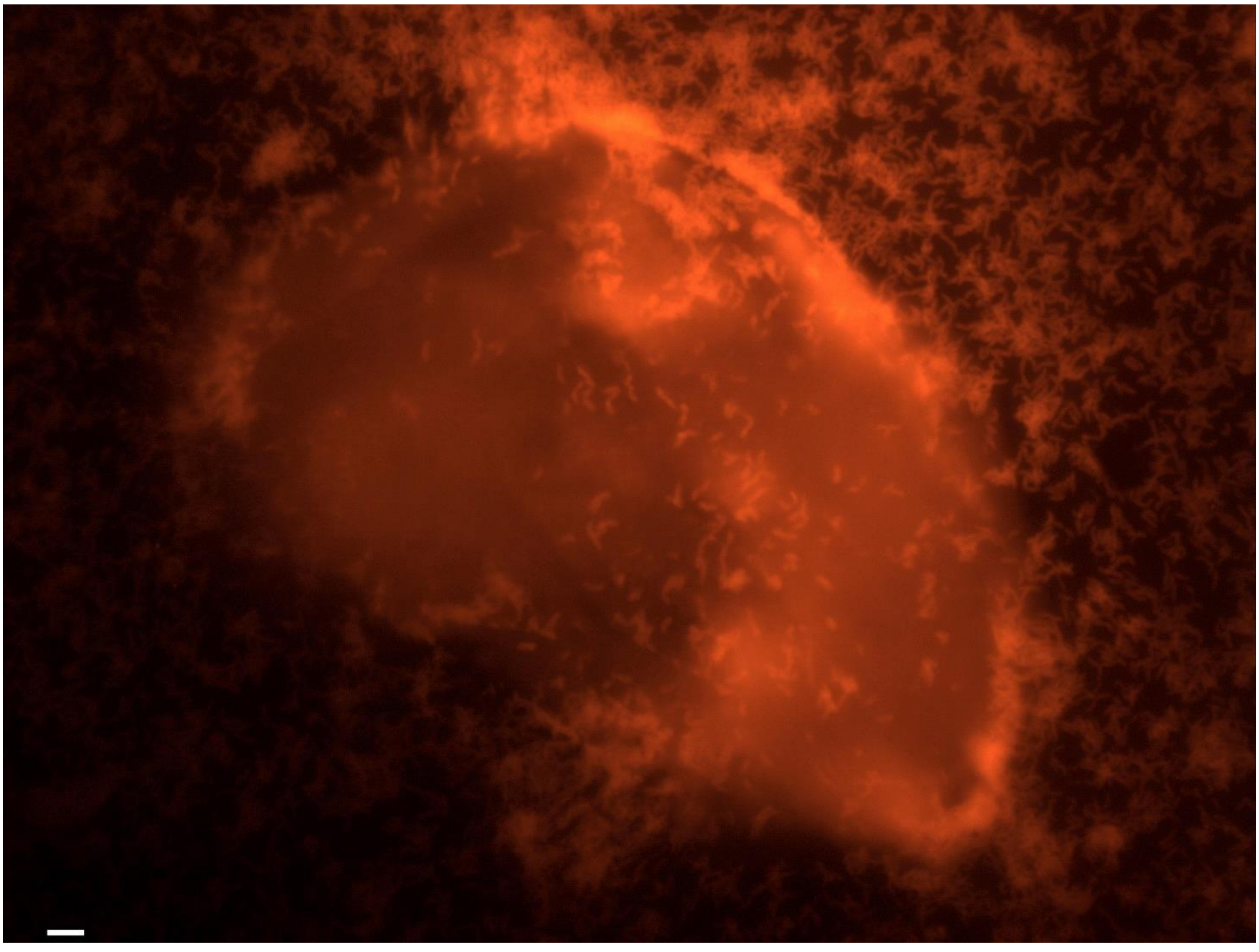

Supplementary Figure S5: investigation on putative production of hydrogen peroxide by *Rheinheimera* sp. EpRS3 strain. Left side: different amounts of hydrogen peroxides were placed on the PB-TSA medium, revealing the appearance of the blue precipitate. White values stand for the nanomoles of H2O2 in each drop. Right side: 5-days growth of *Rheinheimera* sp. EpRS3 strain on PB-TSA medium.

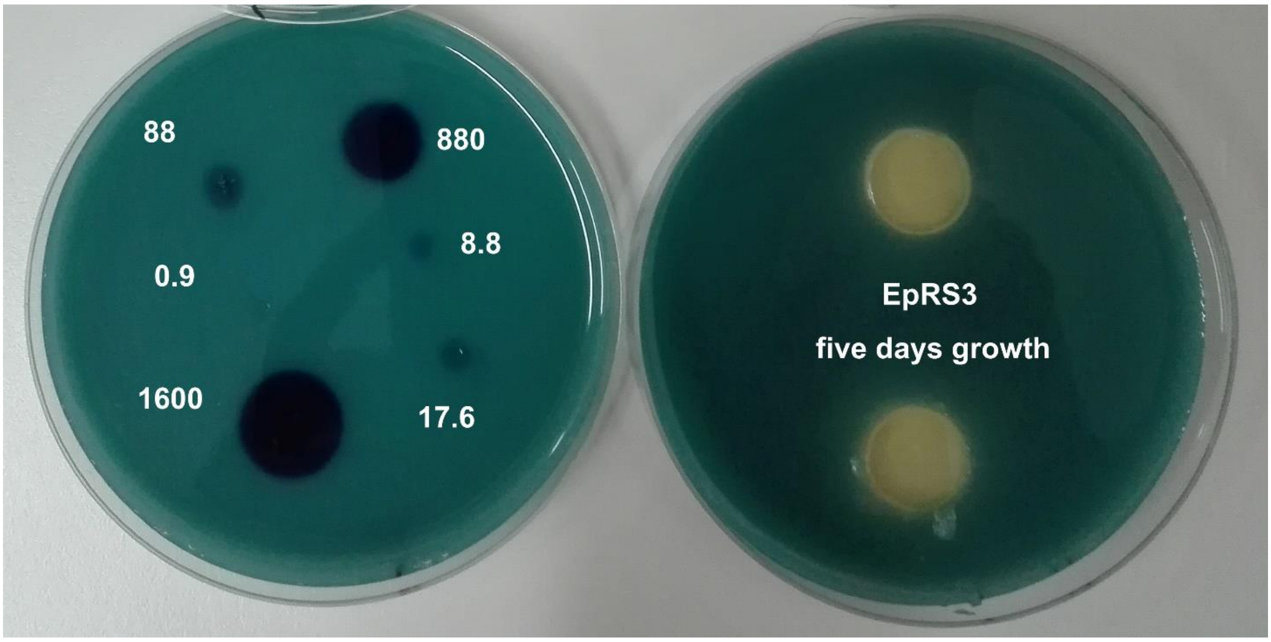

Supplement: Supplementary file 1 [file Data_Sheet_1.PDF]
