# Supplementary material for: Harmful Effect of Rheinheimera sp. EpRS3 (Gammaproteobacteria) Against the Protist Euplotes aediculatus (Ciliophora, Spirotrichea): Insights Into the Ecological Role of Antimicrobial Compounds From Environmental Bacterial Strains
Source: Front Microbiol. 2019 Mar 22;10:510. doi: 10.3389/fmicb.2019.00510 (PMC6457097; doi:10.3389/fmicb.2019.00510)
Supplement: Supplementary file 2 [file Data_Sheet_2.pdf]

Supporting Information

Harmful effect of *Rheinheimera* sp. EpRS3 (*Gammaproteobacteria*) against the protist *Euplotes aediculatus* (Ciliophora, Spirotrichea): insights into the ecological role of antimicrobial compounds from environmental bacterial strains

Carolina Chiellini, Chiara Pasqualetti, Olivia Lanzoni, Camilla Fagorzi, Chiara Bazzocchi, Renato Fani, Giulio Petroni, and Letizia Modeo

Table S1: Description of the five experimental treatments applied to *Euplotes aediculatus* sub-cultures

	Treatment	Amount of treatment for each ciliate sub-culture (1 ml)	Treatment description	Treatment purpose
A	C+	100 µl	Sterile Tryptic Soy Broth (TSB) medium (positive control)	To assess any possible effect on ciliates of the bacteria growth medium
B	C-	---	Absence of any treatment (negative control)	To assess that the experimental conditions (e.g. cell manipulation) do not affect the growth and survival of ciliates
C	Supernatant	100 µl	2 ml of filtered *Rheinheimera* sp. EpRS3 liquid culture (0.2 µm), cell-free supernatant.	To assess the effect on ciliates of possible bioactive molecule(s) only, dissolved in the growing medium, without living bacterial cells
D	Tq	100 µl	*Rheinheimera* sp. EpRS3 cells grown in Tryptic Soy Broth (TSB) medium	To assess the effect on ciliates both of EpRS3 cells grown in the liquid medium and any possible bacterial bioactive molecule(s) produced during overnight growth
E	Pellet	100 µl	2 ml of centrifuged *Rheinheimera* sp. EpRS3 liquid culture and pellet resuspended in saline solution (0.9% NaCl in dH_2O)	To assess the effect on ciliates of bacterial cells only, without possible bioactive molecule(s) dissolved in the growing medium
